# Effects of whole-body vibration or resistive-vibration exercise on blood clotting and related biomarkers: a systematic review

**DOI:** 10.1038/s41526-023-00338-4

**Published:** 2023-12-06

**Authors:** Lucrezia Zuccarelli, Giovanni Baldassarre, Andrew Winnard, Katie M. Harris, Tobias Weber, David A. Green, Lonnie G. Petersen, Tovy Haber Kamine, Lara Roberts, David S. Kim, Danielle K. Greaves, Roopen Arya, Jonathan M. Laws, Antoine Elias, Jörn Rittweger, Bruno Grassi, Nandu Goswami

**Affiliations:** 1 Department of Medicine, University of Udine, Udine, Italy; 2 Space Biomedicine Systematic Review Methods, Wylam, UK; 3 Faculty of Medicine, Memorial University of Newfoundland, St. John’s, Newfoundland Canada; 4 Space Medicine Team, European Astronaut Centre, European Space Agency (ESA), Cologne, Germany; 5 KBR GmbH, Cologne, Germany; 6 Centre for Human and Applied Physiological Sciences, King’s College London, London, UK; 7 Department of Biomedical Sciences, University of Copenhagen, Copenhagen, Denmark; 8 Department of Aeronautics and Astronautics, Massachusetts Institute of Technology, Cambridge, MA USA; 9 Division of Trauma, Acute Care Surgery, and Surgical Critical Care, Baystate Medical Center, Springfield, MA USA; 10 Kings College Hospital, NHS Foundation Trust, London, UK; 11 Department of Emergency Medicine, Faculty of Medicine, University of British Columbia, Kelowna, Canada; 12 Department of Kinesiology and Health Sciences, Faculty of Health, University of Waterloo, Waterloo, Ontario Canada; 13 Department of Vascular Medicine, Sainte Musse Hospital, Toulon La Seyne Hospital Centre, Toulon, France; 14 Institute of Aerospace Medicine, German Aerospace Center (DLR), Cologne, Germany; 15 Division of Physiology, Otto Löwi Research Center for Vascular Biology, Immunity and Inflammation, Medical University of Graz, Graz, Austria; 16 Mohammed Bin Rashid University of Medicine and Applied Health Sciences, Dubai, UAE

**Keywords:** Diagnostic markers, Cardiovascular diseases

## Abstract

Whole-body vibration (WBV) and resistive vibration exercise (RVE) are utilized as countermeasures against bone loss, muscle wasting, and physical deconditioning. The safety of the interventions, in terms of the risk of inducing undesired blood clotting and venous thrombosis, is not clear. We therefore performed the present systematic review of the available scientific literature on the issue. The review was conducted following the guidelines by the Space Biomedicine Systematic Review Group, based on Cochrane review guidelines. The relevant context or environment of the studies was “ground-based environment”; space analogs or diseased conditions were not included. The search retrieved 801 studies; 77 articles were selected for further consideration after an initial screening. Thirty-three studies met the inclusion criteria. The main variables related to blood markers involved angiogenic and endothelial factors, fibrinolysis and coagulation markers, cytokine levels, inflammatory and plasma oxidative stress markers. Functional and hemodynamic markers involved blood pressure measurements, systemic vascular resistance, blood flow and microvascular and endothelial functions. The available evidence suggests neutral or potentially positive effects of short- and long-term interventions with WBV and RVE on variables related to blood coagulation, fibrinolysis, inflammatory status, oxidative stress, cardiovascular, microvascular and endothelial functions. No significant warning signs towards an increased risk of undesired clotting and venous thrombosis were identified. If confirmed by further studies, WBV and RVE could be part of the countermeasures aimed at preventing or attenuating the muscular and cardiovascular deconditioning associated with spaceflights, permanence on planetary habitats and ground-based simulations of microgravity.

## Introduction

Whole-body vibration (WBV) is frequently used as a training and/or rehabilitative approach. This modality has been utilized in subjects or patients who cannot do other forms of exercise, such as children, patients with spinal cord injury, patients with very limited aerobic performance and patients with limited compliance due to physical or behavioral limitations^1–3^. In WBV, mechanical oscillations are transferred to the human body by standing on a vibrating plate. In healthy and patient groups WBV has been shown to obtain varying degrees of improvement of neuromuscular function, bone density, muscle mass, muscle strength, and power^4^.

Resistive exercise can be combined with WBV. Because of the synergistic effects, this combination appears to offer multiple beneficial advantages such as increased muscle activity and neuromuscular feedback^5,6^. WBV and resistive vibration exercise (RVE) have been used as countermeasures to prevent the impairments of skeletal muscle function, the loss of muscle mass, muscle force and bone mass described in terrestrial spaceflight analogs, such as bed rest studies^7–9^. Exposure to microgravity and the space environment results in profound multi-system adaptations/impairments, characterized by both short- and long-term changes, including an enhanced coagulation state in the cephalad venous system due to changes in venous flow, jugular vain pressure and endothelial damage^10^, reductions in maximum oxygen uptake (V̇O_2_max), impairments of endothelial/microvascular^11^ and mitochondrial function^12^ and skeletal muscle oxidative metabolism^13,14^, reduced muscle size and strength and bone mineral density^15^. RVE was used in the Berlin Bed Rest studies^16–18^, in which the intervention resulted in beneficial effects on bone loss, bone metabolism, muscle mass loss and muscle contractile capacity. In these studies the effects of RVE on cardiovascular deconditioning were not assessed, and were presumed minor. On the other hand, interesting vascular effects were observed: RVE attenuated the diameter decrease of leg conduit arteries^19^, prevented completely (carotid artery) or partially (superficial femoral artery) the increase in arterial wall thickness^20^, and abolished the marked increase in flow-mediated dilation and the decrease in baseline diameter of the superficial femoral artery normally associated with prolonged bed rest^21,22^.

In terms of safety and side effects of WBV and RVE, apart from reports of itching and erythema^23,24^, studies are lacking. A potential risk could be related to the development of deep vein thrombosis^25^, a condition which was recently brought to the attention of the space medicine community following an incidental finding of a persistent asymptomatic obstructive left internal jugular venous thrombus in a single crewmember of the International Space Station^26^. Whereas WBV and RVE could act, as other types of exercise do, in the direction of favoring vascular health and preventing undesired clotting, the question could be asked whether and how vibration interferes with blood flow and in particular with venous return. Clearly, in the presence of an impediment to flow leading to stagnation or even retrograde flow, then this would constitute a cause for concern. Power doppler ultrasound measurements suggest that vibration at frequencies between 10 and 30 Hz increases blood flow velocity, probably to an extent that is commensurate with the metabolic demand or even above it^27^. Moreover, near-infrared spectroscopy (NIRS) measurements have demonstrated that vibration extrudes venous blood out of the vibrated muscles during a 30–60-s period^28^, an effect that seems to be depending on the alignment of the main vibration axis and the vessels with the gravity vector^29^. When this alignment does not occur, and/or when normal vasomotility is impaired, retrograde and disturbed flow patterns may lead to an increased risk of undesired clotting and venous thrombosis. Venous nd arterial flow patterns during RVE and WVB and the potential link to an increased risk of developing venous thrombosis are largely unexplored.

Another potential question to be considered is the magnitude of stresses and strains that are caused by vibration. A biomechanical study that has assessed 4 Hz vibration-induced contractile element length changes has reported 1% elongation of the muscle-tendon complex length, and that half of the absolute elongation occurred within the muscles^30^. For comparison, the muscle-tendon complex undergoes elongations in the order of 5% and of 10% during squat jumping and hopping, respectively, which are substantially greater strains than those reported for vibration. Another factor to be considered are shear strains and shear stresses exerted between endothelium and blood. As long as the accelerations stay within the “physiological” range (as e.g., in running), one would expect to see the known physiological endothelial reactions. However, vibration platforms are often used with peak accelerations >10 g, and up to twofold resonant amplitude amplification has been reported in the ankle^31^, suggesting the potential to elicit endothelial shear stresses that are greater than observed during other types of movement or exercise. From this, a potential risk of damage to the vessel wall and endothelium may arise, possibly leading to inflammation, altered redox balance, increased risk of undesired clotting and venous thrombosis. The issue is largely unexplored.

The present systematic review was therefore performed with the aim of identifying and evaluating the effects of RVE and WBV on blood clotting and thrombosis formation in healthy subjects. If proven to be effective and safe, WBV and RVE could be relevant in the future for space explorations, as they could be implemented during spaceflights and/or inside human habitats during sustained planetary missions, in which the operational constraints will be more severe than on the International Space Station^32^. As pointed out in the “Gap analysis and research recommendations” section in the review by Harris et al.^25^, an assessment of the effects of WBV and RVE interventions on the risk of undesired clotting and venous thrombosis during spaceflight and in ground-based analogs is a research gap, which the present systematic literature review seeks to fill.

## Methods

### Identification and protocol

A systematic review of the literature was conducted following the guidelines defined by the Space Biomedicine Systematic Review Group^33^ and the PRISMA (Preferred Reporting Items for Systematic Reviews and Meta-Analyses) Guidelines for Systematic Review^34^. Initial pre-scoping was performed to determine appropriate search terms that would capture an adequate number of papers to reach knowledge saturation. The members of the ESA Topical Team on Venous Thromboembolism (VTE) contributed with their expertise to ensure that all appropriate terms were included^25,35^.

### Eligibility criteria

An extensive literature search was performed using recognized life science and biomedical electronic databases and by manually searching reference lists of the articles which specifically investigated the effects of WBV or RVE on blood coagulation and related biomarkers, as well as on related variables such as blood vessels, blood flow and endothelial function. No language, publication date, or publication status restrictions were imposed. This search was applied to the following electronic databases: PubMed, Web of Science, Cochrane. The latest search was performed during April 2022.

The Population, Interest, Control, and Outcome (PICO) table used to define the present research criteria is shown in Table 1, along with the keywords. The population to be studied was chosen as “healthy subjects”; studies on diseased populations or animal studies were excluded. The interventions were subdivided into “short-term” (single session) or “long-term” (more than one session) WBV or RVE. The biomarkers and variables directly or indirectly associated with a risk of undesired clotting and thrombi formation, to be evaluated in the analysis, included: blood levels of nitrites, nitrates, prostacyclin (6-keto), von Willebrand factor, endothelin, hyaluronan, syndecan-1; levels of heparan sulfates, heparanase, endocan, prothrombin fragment, thrombin–antithrombin III complex; glycocalyx integrity; fibrinogen synthase rate, clot formation time, clotting time, extrinsic pathway thromboelastometry, fibrinogen thromboelastometry, international normalized ratio, maximal clot firmness, thromboelastometry. The outcomes to be considered were: molecular, hematological, functional and clinical biomarkers of undesired clotting and thrombi formation; symptoms; evidence from imaging or functional studies related to the presence or the risk of undesired clotting and thrombi formation (see Table 1). The relevant context or environment of the studies to be considered was chosen as “ground-based environment”; space analogs or diseased conditions were not included in this review.

**Table 1:** Elements of the search strategy.

Category	Specific category	Keywords	Search Number	Search Mask
Population	Inclusion criteria	“human” OR “humans” OR “women” OR “woman” OR “man” OR “men” OR “female” OR “male” OR “adult”	1	All fields
Interest	Exercise intervention	“Whole body vibration” OR “resistive vibration exercise” OR “WBV” OR “RVE”	2	All fields
	Venous thromboembolism	“venous thrombo*” OR “VT” OR “VTE” OR “clot” OR “embol*” OR “DVT” OR “Stasis” OR “blood stasis” OR “blood flow” OR “venous system” OR “venous physiology” OR “venous pathology” OR “venous function” OR “venous flow” OR “venous pathology” OR “venous pathophysiology” OR “venous disease” OR “venous pressure” OR “venous circulation” OR “venous hemodynamics” OR “vessel damage” OR “vessel injury”	3	All fields
	Endothelial function	“endothel*” OR “intima”	4	All fields
	Coagulation	“coagulation” OR “clotting” OR “coagulation cascade” OR “hemostasis” OR “hemostasis” OR “thrombosis” OR “thrombus” OR “coagulopathy” OR “thromboembolism”	5	All fields
Control	N/A	N/A		N/A
Outcome	Structural biomarkers	“structural biomark*” OR “vessel wall thickness” OR “wall structural change*” OR “venous thrombogenesis” OR “Cerebral Blood Volume”	6	All fields
	Venous mechanical properties	“venous compliance” OR “wall extensibility” OR “veins” OR “vasoconstriction” OR “vasodilation”	7	All fields
	Venous flow properties	“venous flow” OR “venous flow direction” OR “venous flow velocity” OR “venous flow volume” OR “Venous blood pressure” OR “venous BP” OR “venous pressure”	8	All fields
	Symptoms	“swelling” OR “edema”	9	All fields
	Circulating biomarkers	“Soluble P-SELECTIN” OR “inflammatory cytokine*” OR “ICAM-1” OR “intercellular adhesion molecule-1” OR “cell-free DNA” OR “interleukin-6” OR “IL-6” OR “IL-8” OR “interleukin-8” OR “IL-10” OR “interleukin-10” OR “p-selectin” OR “intercellular adhesion molecule-1” OR “cell-free nucleic acids”	10	All fields
	Endothelial markers	“endothelial marker*” OR “Tissue Factor” OR “TF” OR “Tissue-plasminogen activator” OR “tPA” OR “thromboplastin”	11	All fields
	Blood cell counts	“blood cell count*” OR “complete blood count” OR “CBC” or “white blood cell*” OR “WBC” OR “red blood cell*” OR “RBC” or “hemoglobin” OR “hemoglobin” OR “Hb” OR “hematocrit” or “hematocrit” OR “Hct” OR “platelet*” OR “blood cell count” OR “hemoglobins” OR “hematocrit”	12	All fields
	Thrombelastometry	“thrombelastomet*” OR “TEM” OR “EXTEM” OR “INTEM” OR “FIBTEM” OR “thromboelastograph*” OR “TEG” OR “Sonoclot” OR “CT” OR “clotting time” OR “CFT” OR “clot formation time” OR “MCF” OR “maximum clot firmness” OR “fibrin clot lysis time”	13	All fields
	Platelet aggregation and adhesion	“platelet aggreg*” OR “platelet adhes*” OR “surface cover*”	14	All fields
	Coagulation times	“coagulation” OR “APTT” OR “activated partial thromboplastin clotting time” OR “partial thromboplastin time” OR “PTT” OR “INR” OR “prothrombin” OR “prothrombin time” OR “PT” “thrombin time” OR “activated whole blood clotting time” OR “ACT” OR “anti-factor Xa” OR “anti-Xa” OR “D-dimer” OR “international normalized ratio” OR “blood coagulation” OR “blood coagulation tests”	15	All fields
	Thrombin generation	“thrombin generat*” OR “prothrombin fragment” OR “F1 + 2” OR “TAT” OR “thrombin antithrombin complex” OR “thrombomodulin” OR “lag time” OR “ETP” OR “endogenous thrombin potential” OR “prothrombin fragment 1.2” OR “antithrombin III-protease complex” OR “Protein C” OR “Thrombomodulin”	16	All fields
	Fibrinolytic values/endothelial activation	“fibrinolytic parameter*” OR “endothelial activ*” OR “t-PA Ag” OR “tissue plasminogen activator” OR “tPA” OR “plasminogen activator inhibitor 1” OR “PAI-1 Ag” OR “TF” OR “tissue factor” OR “EndoPAT” OR “RHI” OR “reactive hyperemia index” OR “ADMA” OR “Asymmetric dimethylarginine” OR “nitric oxide” OR “NO” OR “microvasculature” OR “Plasminogen Activator Inhibitor 1”	17	All fields
	Procoagulatory factors	“procoagulation factor*” OR “F II” OR “factor II” OR “F VII” OR “factor VII” OR “F VIII” OR “factor VIII” OR “VWF” OR “von Willebrand factor” OR “Fibrinogen” OR “microparticle*” OR “Blood Coagulation Factors”	18	All fields
	Anticoagulatory factor	“anticoagulation factor*” OR “protein S” OR “antithrombin” OR “TFPI” OR “tissue factor pathway inhibitor” OR “Blood Coagulation Factor Inhibitors”	19	All fields
	Exclusion	“cancer” OR “tumor” OR “malignanc*” OR “neoplasm*” OR “COVID-19” OR “SARS CoV 2” OR “Coronavirus” OR “pregnant” OR “patient” OR “children” OR “bone” OR “osteopor*” OR “chronic obstructive pulmonary” OR “disease” OR “COPD” OR “hepatitis” OR “animal” OR “mice” OR “mouse”	20	All fields
Search strategy		#1 AND #2 NOT #20 OR #3 OR #4 OR #5 OR #6 OR #7 OR #8 OR #9 OR #10 OR #11 OR #12 OR #13 OR #14 OR #15 OR #16 OR #17 OR #18 OR #19		

Two members of the project team independently conducted the search strategy (L.Z. and G.B.). Participants of any age and sex were included. All retrieved records were screened by title and abstract by two reviewer authors independently (L.Z. and G.B.). The review authors (L.Z. and G.B.) rated each study using the classifications “relevant”, “irrelevant” or “unsure”. Only retrieved records that received the label “relevant” or “unsure” were full-text screened. Selected articles were then classified into two different categories (i.e., blood markers and functional/haemodynamic outcomes). Disagreements between reviewers were resolved via discussion until a consensus was found and all reasons for exclusion from the study were recorded.

### Reporting summary

Further information on research design is available in the Nature Research Reporting Summary linked to this article.

## Results

### Characteristics of included studies

**Fig. 1:**
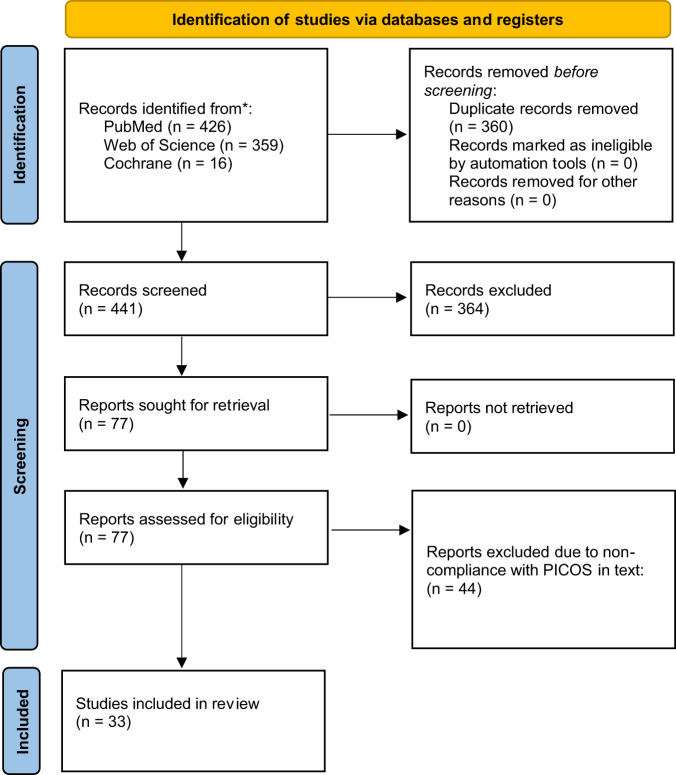
Search and screening strategy based on PRISMA 2020 flow diagram for new systematic reviews. *Papers excluded due to lack of adherence to PICO.

The systematic search retrieved 801 articles which were screened for duplicates and appropriateness using the Rayyan online platform^36^. After the initial screening, 77 articles were retained, 33 of which were included in the study. The most common reason for papers to be excluded was that vibrations were applied only on small parts of the body and because of wrong outcomes. The full screening flow is shown below in Fig. 1.

Twenty-five papers were categorized as “short-term intervention” WBV or RVE, and 8 as “long-term intervention” WBV or RVE studies. The characteristics of the studies considered eligible for inclusion are summarized in Tables 2 and 3, respectively. All 33 studies included in this review contained original data and were published in English.

**Table 2:** Characteristics of “short-term intervention” studies.

Author (year) [reference]	Study design	Sample size (*n*) [males]	Population	Age (years)	Intervention type	Experimental protocol	Vibration platform	Interval measurement	Parameters	Main results
Tsung-Cheng & Zong-Yan et al.^45^	Longitudinal study	16 [16]	Inactive	20^a^	RVE	10 sets (each with 1 min WBV and 1 min rest) of intermittent static squat position (100° knee flexion)	Brand: BH YT18 (Taipei, Taiwan). Movement principle: NA. Frequency: 26 Hz. Peak-to-peak amplitude: 4 mm.	Pre- and post activity	Willebrand factor (vWF) levels	vWF: ↑
Betik et al.^52^	Longitudinal study	11 [5]	Adults	33 ± 2^b^	WBV and RVE	3 min of WBV	Brand: Galileo Fitness, Novotech (Pforzheim, Germany). Movement principle: simultaneous side-alternating. Frequency: 15 Hz. Peak-to-peak amplitude: 6-8 mm.	Pre, post activity and 3-min post-intervention recovery period	Femoral arterial blood flow and thigh muscle microvascular blood flow	Femoral arterial blood flow: ↑ Muscle microvascular blood flow: ↑
Jawed et al.^37^	Randomized control trials	11 [11]	Young and old	24–55^c^	WBV and RVE	Three activities: (1) 8 × 60s (120 s rest) standing only on a vibrating platform (WBV), (2) 8 × 60s dynamic leg squats exercise (sq) (RE), and (3) 8 × 60s dynamic leg squats on a vibrating platform (RVE). Subjects squatted to 90° knee flexion. 15 repetitions of leg squats per bout (120 repetitions total). Subject carried an additional 15% of their body weight to increase load	Brand: Power Plate my3 (Northbrook, IL, USA). Movement principle: vertical displacement. Frequency: 35 Hz. Peak-to-peak amplitude: 4 mm.	Pre- and post activity	Blood pressure, circulating stem/progenitor cell (CPC), mononuclear cells (MNC), plasma vascular endothelial growth factor (VEGF), interleukin-6, (IL-6) and tumor necrosis factor-alpha (TNF-α), interleukin-10 (IL-10) cytokines. Blood count markers (hemoglobin, red blood cells, white blood cells)	CPC: ↑ in young VEGF: ↑ TNF-α: ↑ IL-6: ↓ IL-10: ↑
Santos et al.^46^	Longitudinal	21[0]	Healthy controls	50 ± 10^b^	RVE	8 × 40s squat exercise. 40s of each series, for a total of 5 repetitions each	Brand: Fitvibe, Gymna Uniphy NV (Bilzen, Belgium). Movement principle: vertical synchronous displacement. Frequency: 40 Hz. Peak-to-peak amplitude: 4 mm.	Pre- and post activity	Plasma oxidative stress markers, thiobarbituric acid reactive substances (TBARS), iron reduction capacity (FRAP), superoxide dismutase antioxidant enzymes activity (SOD), and catalase (CAT)	TBARS: ↔ FRAP: ↔ SOD: ↑ CAT: ↑
Chih-Min et al.^41^	Crossover design	10 [10]	Inactive	21 ± 1^b^	RVE	10 × 60s isometric squat position, with 1–2 min of rest between sets	Brand: BH YT18 (Taipei, Taiwan). Movement principle: NA. Frequency: 26 Hz. Peak-to-peak amplitude: 4 mm.	Pre-, post- and 15 and 30 min post-intervention	VEGF	VEGF: ↔
Karabulut et al.^57^	Longitudinal	8 [8]	Healthy	23 ± 2.2^b^	RVE	8 × 45s push up (elbows 110°) and 10x60s isometric squat (knees at 110° of flection)	Brand: Power Plate Pro5 Airdaptive (Northbrook, IL, USA). Movement principle: NA Frequency: 30 Hz. Peak-to-peak amplitude: NA.	Pre- and 10 and 40 min post-intervention	Arterial stiffness (pulse wave analysis -radial artery), blood pressure (BP)	Arterial stiffness: ↓
Dipla et al.^50^	Controlled trial	12 [0]	Premenopausal lean controls	32–42^c^	WBV	6 min WBV. The control protocol was the same with no vibration.	Brand: Galileo Fitness, Novotech (Pforzheim, Germany). Movement principle: simultaneous side-alternating. Frequency: 25 Hz. Peak-to-peak amplitude: 6 mm.	Pre-, post activity and 4 min post activity	Beat-by-beat BP, systemic vascular resistance	BP: ↑ Systemic vascular resistance: ↓
Menendez et al.^53^	Control randomized studies	13 [13]	Physically active	21 ± 1^b^	RVE	10 × 60 s ON + 60 s off isometric squat position	Brand: Galileo Home, Novotech (Pforzheim, Germany). Movement principle: simultaneous side-alternating. Frequency: 26 Hz. Peak-to-peak amplitude: 5 mm.	Pre-, during and 5 min post activity	Popliteal arterial blood velocity	Popliteal arterial blood velocity: ↑
Robbins et al.^66^	Longitudinal	20 [12]	Healthy	24 ± 3^b^	WBV	5 × 60 s of vibration (60 s on 60 s off), standing position	Brand: Power Plate Pro6 (London, UK). Movement principle: vertical displacement. Frequency: 40 Hz. Peak-to-peak amplitude: 1.9 mm.	Pre- and every minute after each set and	Peripheral venous function, blood flow velocity in the dorsalis pedis artery, and blood pressure	Blood flow velocity: ↑ BP: ↔
Yarar-Fisher et al.^51^	Longitudinal	10 [10]	Healthy controls	48 ± 8^b^	WBV	3–6 min WBV	Brand: WAVE Manufacturing (Windsor, Canada). Movement principle: synchronous vertical displacement. Frequency: 30, 40 and 50 Hz. Peak-to-peak amplitude: ∼ 2 mm.	Pre-, during and post activity	Mean arterial pressure (MAP) and Total hemoglobin (Thb)	MAP: ↔ Thb: ↑
Beijer et al.^40^	Randomized control trial	26 [26] 13 [13] only for RVE	Healthy	26 ± 1^b^	RVE	The exercise consisted of squats and heel raises, 1-min break. The first and second sets were composed of 8 squats and 12 calf raises and in the third set, maximum number of repetitions for squats and calf raises were performed. Participants trained with weights	Brand: Galileo Fitness, Novotech (Pforzheim, Germany). Movement principle: simultaneous side-alternating. Frequency: from 20 to 40 Hz with increments of 5 Hz during the last two weeks. Peak-to-peak amplitude: 6 mm.	Pre- and 2–5– 15–35–75 min post activity	Circulating levels of matrix metalloproteinases (MMP) -2 and -9, Vascular Endothelial Growth Factor (VEGF) and endostatin. Proliferative effect of serum-treated human umbilical vein endothelial cells in vitro	MMP-2: ↔ MMP-9: ↔ VEGF: ↔ Endostatin: ↔
Games & Sefton^54^	Longitudinal	14 [5]	Healthy	22 ± 2^b^	WBV	5 min WBV	Brand: WAVE Manufacturing (Windsor, Canada). Movement principle: synchronous vertical displacement. Frequency: 50 Hz. Peak-to-peak amplitude: 2 mm.	Pre- and 0–5–10 –15–20 min post activity.	Thb	Thb: ↑
Li et al.^67^	Randomized trial	12 [12]	Healthy	26 ± 2^b^	WBV	3 × 20 min WBV. Each at different intensities	Brand: NA Movement principle: vertical displacement. Frequency: 3, 4.5, and 6 Hz. Peak-to-peak amplitude: NA.	Pre and post activity	Wavelet analysis of Thb (muscle oscillations)	Wavelet analysis of Thb: ↓
Robbins et al.^68^	Controlled trial	20 [14]	Healthy	29 ± 10^b^	RVE	10 × 15 heel raises at 1 Hz with and without vibration	Brand: Power Plate Pro6 (Northbrook, IL, USA). Movement principle: vertical displacement. Frequency: 40 Hz. Peak-to-peak amplitude: 1.9 mm.	Pre, during and post activity	Thb	Thb: ↓
Sanchez-Gonzalez^69^	Crossover study	20 [9]	Healthy	22 ± 3^b^	WBV	1 × 10 WBV or no WBV	Brand: NA Movement principle: NA Frequency: 25 Hz. Peak-to-peak amplitude: 2 mm.	Pre and after 3 min and 30 min after intervention	Radial waveforms, augmentation index (Aix). Brachial blood pressure.	Aix: ↓ BP: ↔
Boyle & Nagelkirik^39^	Randomized control trial	20 [20]	Healthy	24 ± 1^b^	WBV and RVE	Each participant performed: exercise, vibration and vibration (15 min) + exercise. 15 min unloaded squatting at rate 20 per minute	Brand: Pineapple Pro (Hollywood, CA, USA). Movement principle: NA. Frequency: 30 Hz. Peak-to-peak amplitude: 1.5 mm.	Pre and post activity	Tissue plasminogen activator (tPA), plasminogen activator inhibitor (PAI-1).	tPA: ↔ PAI-1: ↔
Coza et al. (2010)^60^	Longitudinal	16 [16]	Healthy	26 ± 5^b^	RVE	The subjects were asked to repeatedly rise on their toes, from a normal standing position, at a rate of 40 repetitions per minute. Each subject performed 67 repetitions per trial (100 s in total)	Brand: Vibra Pro 5500, (Colton, CA, USA). Movement principle: vertical displacement. Frequency: 16 Hz. Peak-to-peak amplitude: 4 mm.	Pre-, during and post activity	total hemoglobin index (nTHI)	nTHI: ↑
Rittweger et al. ^38^	Longitudinal	10 [10]	Healthy	29 ± 4^b^	WBV and RVE	Subjects performed WBV and dynamic shallow squatting exercise at comparable levels of oxygen uptake for 3 min	Brand: Galileo Fitness, Novotech (Pforzheim, Germany). Movement principle: simultaneous side-alternating. Frequency: 25 Hz. Peak-to-peak amplitude: 4-6 mm.	Pre-, during and post activity	tHb and VEGF	tHb: ↔ VEGF: ↔
Lytho et al.^27^	Control trial	9 [9]	Healthy	22 ± 4^b^	RVE	12 × 60s squatting with WBV and 2 × 60s squatting without vibration	Brand: Galileo 900, Novotech (Pforzheim, Germany). Movement principle: simultaneous side-alternating. Frequency: 5–30 Hz Peak-to-peak amplitude: 2.5-4.5 mm.	Pre-, during and at 5- 10- 15- 30- 45- 75- post activity	Diastolic pressure, systolic and diastolic diameters of common femoral artery and blood cell velocity	BP: ↔ Femoral artery blood flow: ↑
Hazell et al.^23^	Randomized control trial	11 [11]	Active	25 ± 3^b^	WBV and RVE	2 groups: 15 × 1 min seated on WBV device (passive) and standing in a semi-squat position (static) both with and without WBV	Brand: WAVE, Whole-body Advanced Vibration Exercise (Windsor, Canada). Movement principle: vertical displacement. Frequency: 45 Hz. Peak-to-peak amplitude: 2 mm.	Pre-, during and 2–5–20 min post activity	MAP, femoral artery blood flow	MAP: ↑ Femoral artery blood flow: ↑
Otsuki et al.^55^	Control trial	10 [10]	Healthy	27 ± 2^b^	RVE	10 × 60s static squat position with 60s recovery with and without WBV	Brand: Power Plate (London, UK). Movement principle: vertical displacement Frequency: 26 Hz. Peak-to-peak amplitude: 2–4 mm.	Pre-, 20- 40- 60- min after both trials.	Blood pressure, brachial-ankle pulse wave velocity (as index of arterial stiffness)	BP: ↔ brachial-ankle pulse wave velocity: ↑
Yue et al. ^70^	Longitudinal study	3 [3]	Healthy	26–45^c^	WBV	Each vibration test lasted about 30 s with about 30 s rest in between. Each vibration test on Galileo and Power Plate was divided into two parts with different body modes: the subject was required to keep his body relaxed in the first 15 s, then to keep his body as stiff as possible in the next 15 s. Each vibration test on Bosco System was divided into three parts with different body modes: the subject was required to stand on tip-toes in the first 10 s, then on the full feet but being relaxed in the next 10 s, and then still on the full feet but being as stiff as possible in the third 10 s	Brand: Three different vibrating devices. Galileo (Novetec, Germany), Bosco System (Nemes, Italy), and Power Plate (Power Plate, Germany). Movement principle: simultaneous side-alternating and vertical displacements. Frequency: 5, 10, 15, 20, 25, 30 Hz. Peak-to-peak amplitude: 1,2,3 mm.	Pre-, during and post activity	systolic blood pressure (sBP), diastolic blood pressure (dBP), mean blood pressure (mBP)	BP: ↔
Cardinale et al.^59^	Randomized control trial	20 [20]	Inactive and active	25 ± 3^b^	RVE	Isometric squatting for 110 s	Brand: Fitwave, Medisport (Italy). Movement principle: NA. Frequency: 30, 40 and 50 Hz. Peak-to-peak amplitude: ± 4 mm.	Pre-, during and post activity	Thb	Thb: ↔
Yamada et al.^58^	Control trial	18 [18]	Healthy	27 ± 6^b^	RVE	3 min squatting with and without WBV	Brand: Galileo 900, Novotech (Pforzheim, Germany). Movement principle: simultaneous side-alternating. Frequency: 15 Hz Peak-to-peak amplitude: 2.5 mm.	Pre-, during and post activity	Changes in muscle thb. Blood pressure in 9 out of 18 sbjs	Thb: ↑
Kershan-Schindle et al.^56^	Longitudinal	20 [12]	Physically active	25–35^c^	RVE	Each position was held for 3 min and the exercise was continued without break between the positions. First set: subjects stood with their legs straight and their forefeet parallel to each other on the platform. Second bout: entire feet standing on the platform and moderately bent knees (60–70°). Third set: same at position two but the legs were rotated externally by about 30° and the knee were bent by about 60–70°	Brand: Galileo 2000, Novotech (Pforzheim, Germany). Movement principle: simultaneous side-alternating. Frequency: 26 Hz Peak-to-peak amplitude: 3 mm.	Pre- and post activity	Blood pressure, relative moving blood volume of gastrocnemius, quadriceps, the arterial blood flow of the popliteal artery.	BP: ↔ arterial blood flow of the popliteal artery: ↑

*RVE* resistive vibration exercise, *WBV* whole-body vibration exercise, *↑* increase, *↓* decrease, *↔* no change.

^a^Mean.

^b^Mean ± standard deviation.

^c^Range.

**Table 3:** Characteristics of “long-term intervention” studies.

Author (year) [reference]	Study design	Sample size (*n*) [males]	Population	Age (years)	Intervention type	Intervention duration	Experimental protocol	Vibration platform	Interval measurement	Parameters	Main results
Tsung-Cheng & Zong-Yan et al.^45^	Randomized trials	16 [16]	Healthy young untrained	20^a^	RVE	8 weeks	WBV group (*n* = 8) 10 sets × 1 min of static squat (100° knee flexion) for 20 min/day, 3 days/week	Brand: BH YT18 (Taipei, Taiwan). Movement principle: NA. Frequency: progressively increased every eight sessions by 4-Hz from 26 to 34 Hz. Peak-to-peak amplitude: 4 mm.	Pre- and post-intervention	Willebrand factor (vWF) levels	vWF: ↓
Jaime et al.^62^	Randomized controlled trials	21 [0]	Healthy postmenopausal women	64–67^c^	RVE	12 weeks	RVE (*n* = 13) full squats, high squats, wide squats (starting from an upright position to 90° and 120° knee flexions), and calf raises. The training volume was increased progressively by increasing the intensity of vibration, the number of sets per exercise (2–3), and the total duration of the training session (20–35 min) and by increasing the external load using a weight vest. Control group (*n* = 8)	Brand: Pro6 AIRdaptive, Health Performance International (Northbrook, IL, USA). Movement principle: NA. Frequency: progressively increased from 24 to 40 Hz. Peak-to-peak amplitude: NA.	Pre- and post-intervention	Arterial stiffness, augmentation index (AIx), augmented pressure (AP), brachial flow-mediated dilation (FMD)	Arterial stiffness: ↓ AIx: ↓ AP: ↓ FMD: ↑
Beijer et al.^61^	Randomized controlled trial	13 [13]	Recreationally active, healthy	26 ± 4^b^	RVE	6 weeks	2–3 times per week. Squat 3 × 8, 8, max reps 80% 1RM. Calf raises 12, 12, max reps with simultaneous whole-body vibrations	Brand: Galileo Fitness, Novotech (Pforzheim, Germany). Movement principle: simultaneous side-alternating. Frequency: 20-40 Hz. Peak-to-peak amplitude: 6-mm.	Pre- and post-intervention	Total hemoglobin (tHb) in gastrocnemius muscle (GM) Number of capillaries around fibers in soleus (SOL) muscle biopsies	tHb: ↑ Number of capillaries: ↑
Rodriguez-Miguelez et al.^49^	Randomized controlled trial	28 [8]	Elderly	71 ± 2^b^	RVE	8 weeks	2 days a week. 4 exercises (static or dynamic exercises including half-squat between 120° and 130° knee angle, deep squat with 90° knee angle, wide-stance squat and calves with a knee angle between 120° and 130°) 2 sets 1–2 reps 30–60 s 20–35 Hz (number and duration of repetitions and vibration frequency were increased weekly)	Brand: Fitvibe, Gymna Uniphy NV (Bilzen, Belgium). Movement principle: vertical displacement. Frequency: 20–35 Hz Peak-to-peak amplitude: 4 mm.	Data were collected during a laboratory session carried out one week before and one week after the 8-weeks training period	mRNA and protein levels of makers involved in the TLR2/TLR4 myeloid differentiation primary response gen 88 (MyD88) and TIR domain-containing adaptor inducing interferon (TRIF)-dependent pathways. Anti-inflammatory cytokines interleukin-10 (IL-10). Plasma TNFα and C-reactive protein levels	mRNA and protein levels of makers involved in the TLR2/TLR4, MyD88, and TRIF-dependent pathways: ↓ IL-10: ↑ TNFα: ↓ C-reactive proteins: ↓
Cristi et al.^48^	Longitudinal	16 [9]	Elderly	81 ± 1^b^	RVE	9 weeks	3 days a week. Lower- and upper-body unloaded static and dynamic exercises (isometric squat, dynamic squat, isometric standing calves, isometric squat + isometric biceps/shoulders). Time under tension 30–60 s	Brand: Fitvibe, Gymna Uniphy NV (Bilzen, Belgium). Movement principle: vertical displacement. Frequency: 30–45 Hz. Peak-to-peak amplitude: 2 mm (progressive increase during weeks).	At baseline and after exercise. Pre- and post-intervention	Markers of inflammation (mRNA and protein levels for C-reactive protein, interleukin-6 [IL-6], interleukin-1β [IL-1β], tumor necrosis factor-α [TNF-α] and interleukin-10 [IL-10])	C-reactive protein, IL-6, IL-1β, TNF-α and IL-10: ↔
Ghazalin et al.^47^	Randomized controlled trial	25 [25]	Healthy young	21^a^	RVE	5 weeks	Three groups: high‑amplitude vibration group (*n* = 10), low‑amplitude vibration group (*n* = 10), and control group (*n* = 5). Whole‑body vibration 3 times a week with amplitudes of 4 (high) and 2 mm (low) and progressive frequencies from 25 Hz with increments of 5 Hz weekly. 2 × 3 reps (30–60 s) 30 s rest of Squat, lunges and deep squat	Brand: Fit Vib (Germany). Movement principle: vertical displacement. Frequency: 25 Hz with weekly increments of 5 Hz. Peak-to-peak amplitude: 2–4 mm.	Pre- and post-intervention	Concentrations of fibrinogen, plasminogen, tissue plasminogen activator (tPA), and plasminogen activator inhibitor‑1 (PAI‑1)	tPA: ↑ (high and low amplitude) PAI‑1: ↓ (high amplitude) ↔ (low amplitude) Fibrinogen, plasminogen: ↔ (high and low amplitude)
Beijer et al.^40^	Randomized controlled trial	13 [13]	Healthy and recreationally active	26 ± 1^b^	RVE	6 weeks	2–3 times per week. Squat 3 × 8, 8, max reps 80% 1RM. Calf raises 12, 12, max reps + simultaneous whole-body vibrations	Brand: Galileo Fitness, Novotech (Pforzheim, Germany). Movement principle: simultaneous side-alternating. Frequency: from 20 to 40 Hz with increments of 5 Hz during the last two weeks. Peak-to-peak amplitude: 6 mm.	At the initial and final exercise sessions of the 6-week training. exercise. Blood was collected one hour prior to exercise, and +2 min, +5 min, +15 min, +35 min and +75 min after exercise	Serum concentrations of angiogenic factors MMP-2, MMP-9, VEGF and endostatin. Endothelial Cell Proliferation (human umbilical vein endothelial cells (HUVEC) in vitro	MMP-2: ↑ MMP-9, VEGF, Endostatin and Endothelial Cell Proliferation: ↔
Weber et al.^63^	Randomized controlled trial	13 [13]	Healthy young	24 ± 3^b^	RVE	6 weeks	2 days a week for the first 2 weeks, from the third week 3 days a week. 3 × 8 reps squats and 3 × 12 reps heel raises, with 60 s rest. In the last set of each exercise, the subjects were asked to perform as many repetitions as possible	Brand: Galileo Fitness, Novotech (Pforzheim, Germany). Movement principle: simultaneous side-alternating. Frequency: from 20 to 40 Hz with weekly increments of 5 Hz. Peak-to-peak amplitude: 6-mm.	Data were collected at baseline, after 1, 3, and 6 weeks of training and 3 months after the last training session	Arterial resting diameter, intima-media thickness and flow-mediated dilation (FMD) in the superficial femoral artery (SFA), the brachial (BA) and the carotid arteries (CA)	SFA resting diameter: ↑ CA wall thickness: ↓ FMD in SFA, BA and CA: ↔

*RVE* resistive vibration exercise, *WBV* whole-body vibration exercise, *1RM* one-repetition maximum, *↑* increase, *↓* decrease, *↔* no change.

^a^Mean.

^b^Mean ± standard deviation.

^c^Range.

All 25 “short-term intervention” studies were published between 2001 and 2020, and included a total of 348 participants (267 males and 81 females with an age range of 20–55 years). Eleven different vibration platforms were utilized; the most common were Galileo Fitness, Novotech (Pforzheim, Germany) which generates vibration by oscillating along the sagittal axis and the vertical sinusoidal device, Power Plate (International Ltd., London, UK) (all vibration platforms utilized in the included studies are reported in Table 2). The frequency and amplitude (peak-to-peak displacement) of the vibration ranged from 5 to 50 Hz and from 1 to 6 mm, respectively (for more details, see Table 2).

Five studies out of 25 investigated the effects of both WBV and RVE. Whole-body vibration studies (*n* = 12) exposed the participants to vibrations from 1 to 20 min. RVE studies (*n* = 18) included isometric and dynamic squats, push-ups, and heel raises with a duration between 30 s and 15 min, or exercise series up to 8 × 60 s (see Table 2). Data of circulating blood markers, functional/hemodynamic markers, and skeletal muscle oxygenation markers were extracted.

The eight “long-term intervention” studies were published between 2013 and 2020 and included a total of 144 participants (97 males and 47 females with an age range of 20–81 years). Five different vibration platforms were utilized. The most commonly utilized instruments were the simultaneous side-alternating whole-body vibration platforms Galileo Fitness, Novotech (Pforzheim, Germany) and the vertical vibration platform, Fitvibe, Gymna Uniphy NV (Bilzen, Belgium). The frequency and amplitude (peak-to-peak displacement) of the vibrations ranged from 20 to 40 Hz and from 2 to 6 mm, respectively (for more details, see Table 3).

No “long-term intervention” study involved WBV alone. The “long-term intervention” studies (*n* = 8) for RVE involved 2–3 sessions of RVE per week for a duration of 5–12 weeks, and the execution of static or dynamic exercises mainly for the lower body (squats and calf raises), for a total of 2–3 sets for ~8–12 repetitions or 30–60 s of exercise (see Table 3).

### Methodological quality of included studies

The studies differed in study design (e.g., age of participants, sex, and type of resistive exercise), mechanical vibration stimulus (e.g., frequency, amplitude, duration of vibration exposure and platform utilized) and measurement intervals. The marked methodological heterogeneity across the studies and the limited number of papers prevented a meta-analysis. Hence, the results are described qualitatively.

### Effects of whole-body vibration or resistive vibration exercise on blood markers—short-term interventions

The main variables related to blood markers in both WBV and RVE studies involved angiogenic and endothelial factors, fibrinolysis and coagulation markers, cytokine levels, and plasma oxidative stress markers (see Table 2).

Jawed et al.^37^ investigated the vascular endothelial growth factor (VEGF) responses after 8 × 60 s in young (i.e., 24 ± 1 years) and old (55 ± 3 years) participants who stood on a vibration platform. An increase in VEGF and in the non-angiogenic circulating stem/progenitor cell (CPC) levels were found in the young participants but not in the elderly, suggesting a selective positive effect on young subjects on the maintenance of vascular health^37^. The study by Rittweger et al.^38^, however, did not confirm these findings, reporting no change in VEGF following 3 min of WBV in young participants (29 ± 4 years). However, these negative results may be due to the poor sensitivity of the ELISA kit used in that study. Jawed et al.^37^ reported an increase in tumor necrosis factor-alpha (TNF-α) which, together with the increases in VEGF, pointed to a pro-angiogenic effect, even if no changes were observed in the angiogenic CPCs and endothelial colony-forming cells following WBV, both in young and old subjects^37^.

Boyle and Nagelkirik^39^ reported no changes in plasminogen activator inhibitor (PAI-1) and in tissue plasminogen activator (tPA) levels, suggesting an unchanged fibrinolytic activity following 15 min of WBV.

Cytokine levels were investigated during WBV by Jawed et al.^37^. Increased levels of anti-inflammatory cytokine (interleukine-10), associated with a decreased level of inflammatory interleukin-6 point toward a reduced inflammatory state^37^.

WBV significantly increased hemoglobin and platelet counts with no effects on white blood cells, red blood cells, hematocrit, and neutrophil levels^37^.

As for RVE, the angiogenic and endothelial factors did not show any further benefit compared to resistive exercise alone (not associated with vibrations)^40,41^. Angiogenic CPCs increased in young participants but not in the elderly during RVE, and not during resistive exercise alone or WBV^37^. Matrix metalloproteinases (MMPs) have been associated with the release and bioavailability of growth factors and seem to play a role in initiating endothelial cell migration and proliferation as well as in physiological angiogenesis^42–44^. Beijer et al.^40^, reported no changes in the circulating levels of matrix metalloproteinases −2 (MMP-2) and −9 (MMP-9) when RVE was compared to resistive exercise alone, suggesting that vibration did not have any further angiogenic stimulus when applied during resistive exercise. No change was found in the serum levels of endostatin between RVE and resistive exercise alone^40^. No change^37,38,41^ or lower levels^40^ of VEGF were found when RVE was compared to resistive exercise alone.

Increases in tPA and decreases in PAI-1 suggested an increased fibrinolytic activity during RVE^39^. Von Willebrand factor (vWF) was increased after RVE suggesting possible vascular dysfunction^45^.

A trend for reduced levels of pro-inflammatory interleukin-6 with unchanged values of TNF alpha and interleukin-10 suggests a reduced inflammatory state following RVE; no differences were observed compared to resistive exercise alone^37^.

RVE increased neutrophil levels, with no effects on hemoglobin, hematocrit, red blood cells, white blood cells, and platelets^37^.

One study investigated the effects of RVE on oxidative stress markers in 21 females^46^. RVE resulted in an increased superoxide dismutase antioxidant enzymes activity (SOD) and catalase (CAT), with no effect on thiobarbituric acid reactive substances (TBARS) and iron reduction capacity (FRAP), suggesting an improved antioxidant function^46^.

### Effects of whole-body vibration or resistive vibration exercise on blood markers—long-term interventions

The main variables related to blood markers in “long-term intervention” RVE studies involved, as in short-term intervention studies, angiogenic and endothelial factors, fibrinolysis and coagulation markers and cytokine levels (see Table 3). No long-term WBV studies were found.

Following RVE, circulating levels of MMP-2 were generally elevated after 6-week of training compared to baseline in young healthy and recreationally active participants; both at rest and post-exercise MMP-2 levels were significantly higher compared to resistive exercise alone^40^. No changes in MMP-9, VEGF or in endothelial cell proliferation were observed after both exercise and vibration interventions^40^. Circulating post-exercise endostatin levels were higher only after intervention with resistive exercise (no vibrations involved)^40^. Therefore, it seems that superimposing a vibration stimulus to resistance exercise might not be beneficial for triggering angiogenic-inducing signaling pathways in skeletal muscle^40^.

Five weeks of a high-amplitude (4 mm) vibration training program caused an increase in tPA and a decrease in PAI-1^47^. Fibrinogen and plasminogen levels showed a decrease, albeit not significant^47^. Low-amplitude vibration training showed an increase in tPA. PAI‑1, fibrinogen and plasminogen slightly decreased, but did not change significantly^47^. No differences between groups (high- *vs*. low-amplitude vibration) in tPA, PAI‑1, plasminogen, and fibrinogen were observed^47^. These results suggest that resistive vibration training positively affects fibrinolytic activity.

Although vWF was increased after short-term intervention RVE, it significantly decreased after 8 weeks of training, suggesting a beneficial effect of RVE training on vascular function in a previously untrained population^45^.

Nine weeks of training with RVE did not alter inflammatory markers (i.e., levels of C-reactive protein, IL-6, IL-1β, TNF-α, and IL-10) in healthy older adults^48^. Rodriguez-Miguelez et al.^49^ reported an improved anti-inflammatory status in elderly subjects after a 8-week RVE training program. More specifically, a reduced mRNA and protein levels of markers involved in the toll-like receptors (TLR2/TLR4) myeloid differentiation primary response gene 88 (MyD88) and TIR domain-containing adaptor-inducing interferon (TRIF)-dependent pathways were reported. Also, plasma concentration of pro-inflammatory C-reactive protein and TNF-α decreased after training, whereas anti-inflammatory cytokine IL-10 were upregulated^49^.

### Effects of whole-body vibration and resistive vibration exercise on functional/hemodynamic markers—short-term interventions

Functional and hemodynamic markers mostly involved blood pressure measurements, systemic vascular resistance, blood flow and arterial stiffness measurements (see Table 2). Heterogeneous results have been reported for blood pressure values during WBV. Dipla et al.^50^ observed an increase in systolic and diastolic blood pressure with WBV, as well as a decrease in systemic vascular resistance in premenopausal women (age: 37 ± 1.5 years)^50^. On the contrary, Jawed et al.^37^, Hazell et al.^23^, and Yarar-Fisher et al.^51^ did not find any change in blood pressure.

Blood flow changes were mostly assessed by eco-doppler in the femoral artery and in the popliteal artery (see Table 2). Betik et al.^52^ investigated the blood flow changes in the femoral artery in response to different vibration frequencies (i.e., 5–7.5–10–12–15 Hz). They observed an increase in blood flow by about fourfold, and the greatest increase was achieved with 12.5 Hz. The increase in blood flow following WBV was also confirmed by Menendez et al.^53^, who observed an increase in peak blood velocity in the popliteal artery. On the contrary, Hazell et al.^23^ did not see any changes in the common femoral artery during 15 × 60 s of WBV. Skeletal muscle oxygenation was mainly evaluated by NIRS. An increase in total hemoglobin was observed during WBV, suggesting vasodilatation^51,54^.

As for RVE, blood pressure measurements, systemic vascular resistance, blood flow and arterial stiffness measurements have been assessed (see Table 2). Blood pressure has been reported to be unchanged in RVE vs. baseline conditions^27,55,56^ and increased^23^. A reduced systemic vascular function (i.e., capacitive [for large artery] and oscillatory [for small artery] arterial compliance) and arterial stiffness have been reported following RVE^55,57^. There is a general agreement among studies indicating an increased blood flow during RVE. An increased blood flow was observed in the femoral^23,27^ and popliteal arteries^53,56^, as well as in muscular blood flow in the calf and thigh^56^. No changes in total hemoglobin (determined by NIRS) have been reported during RVE^38,58,59^ in vastus lateralis and in gastrocnemius. In contrast, Yamada et al.^58^ and Coza et al.^60^ observed an increase in total hemoglobin during RVE.

### Effects of whole-body vibration and resistive vibration exercise on functional/hemodynamic markers—long-term interventions

Six weeks of training with RVE determined positive effects on vascular function, as shown by a larger reactive hyperemia (greater increase in total hemoglobin [tHb] evaluated by NIRS over baseline in the recovery period after a set of calf raises) and an increased blood volume (larger absolute tHb value at baseline and both during and after exercise) compared to resistance exercise alone^61^. In healthy postmenopausal women (64 ± 1 years), both RVE and resistive exercise induced similar improvements in brachial artery (BA) endothelial function (i.e., increased FMD) after 12 weeks of training^62^. On the other hand, Weber et al.^63^ did not find any changes in FMD after both interventions (RVE and resistive exercise) in any of the investigated arteries (i.e., superficial femoral artery [SFA], BA and carotid arteries [CA]) in young healthy subjects following 6 weeks of training.

Some positive structural adaptations were found after vibration training. Beijer et al.^61^ reported an increased number of capillaries around fibers in the soleus muscle after 6 weeks of both RVE and resistive exercise in recreationally active and young men. Moreover, an increased SFA resting diameter and a reduced arterial wall thickness in CA were described after 6 weeks of resistive exercise, with and without vibration^63^.

Improvements in indices of wave reflection and cardiac pulsatile load (i.e., increased pulse pressure amplification, reduced augmentation index and augmented pressure) were also reported in healthy postmenopausal and normal weight women after 12-week RVE training program, but not after only resistive exercise intervention alone^62^. Therefore, RVE training may have a greater benefit in preventing cardiovascular events compared to resistive exercise alone.

## Discussion

The safety of WBV and RVE, in terms of the risk of inducing undesired blood clotting and venous thrombosis, is not clear. We therefore performed the present systematic review of the available scientific literature on the issue. The limited available evidence suggests neutral or potentially positive effects of short- and long-term interventions with WBV and RVE on variables related to blood coagulation, fibrinolysis, inflammatory status, oxidative stress, cardiovascular, microvascular, and endothelial functions. No significant warning signs towards an increased risk of undesired clotting and venous thrombosis deriving from WBV or RVE were identified.

In total, 33 studies that investigated the effects WBV and RVE on both clotting and thrombosis formation in healthy participants were included. Two broad categories of biomarkers were identified, which were circulating blood and functional/hemodynamic markers. All studies were grouped under these two classifications and further subdivided into short- and long-term interventions.

As mentioned above, the marked methodological heterogeneity across the selected studies and the limited (very limited for several aspects) number of available studies prevented a formal meta-analysis of studies. In particular frequency, amplitude and movement principle were found to be very heterogeneous between the included studies (see Tables 2 and 3). Since the effects of WBV and RVE are strongly dependent upon the parameters that characterize mechanical vibration, such as the frequency and amplitude of the vibration as well as the duration of vibration exposure^27,64^ the results are described in qualitative terms, and they are intrinsically associated with a significant degree of uncertainty. Overall, the analysis stresses the need for further research on the topic.

Data on coagulation factors following short-term intervention WBV or RVE are very scarce. Only one study^45^ observed increased levels of von Willebrand factor after RVE, suggesting a possible vascular dysfunction. In the same study, however, levels of the von Willebrand factor significantly decreased after eight weeks of RVE training. If present, therefore, negative effects of RVE on blood coagulation factors were only transitory, and a positive effect may be present following long-term exposure. These results are in agreement with studies which show an increased risk of thrombotic events after acute exercise, particularly in sedentary individuals, but a decreased risk after regular physical activity^65^. Fibrinolytic activity was unchanged following short-term exposure to WBV^39^, whereas long-term exposure^47^ RVE increased fibrinolytic activity.

Overall, it can be concluded that the few studies available do not suggest the presence of an increased risk of blood coagulation, or of impairments of fibrinolytic activity in subjects exposed to WBV or RVE. Actually, short- or long-term intervention with RVE may exert positive effects on these functions.

In terms of angiogenesis and endothelial function, conflicting results^37,38^ are present in the literature as far as VEGF and CPC levels following short-term exposure to WBV. On the other hand, short-term RVE had positive effects on these factors, although not greater than those obtained with resistive exercise alone (not associated with vibrations)^40,41^. An exception might be represented by MMPs, molecules considered to be essential for extracellular matrix degradation and physiological angiogenesis, for which higher values following long-term intervention with RVE were observed compared to those described following resistive exercise alone^40^.

Studies dealing with cytokine levels concur in indicating a reduced inflammatory state following short-term intervention WBV (see e.g., 37). Following short-term RVE the positive effects on the inflammatory state were not greater compared to those obtainable with resistive exercise alone^37^. Unchanged^48^ or improved^49^ inflammatory markers were described following long-term RVE interventions. Considering the pivotal role played by inflammation in promoting undesired coagulation, the effects of WBV and RVE on the latter seem to be either neutral or positive. The same concept could be applied to antioxidant function, which was found to be improved following short-term exposure to RVE^46^.

Increases in blood flow during short-term intervention WBV^52,53^ or RVE have been described. By preventing blood stasis, slow, stagnant or retrograde blood flow and by enhancing shear stress, increases in blood flow may favor the prevention of undesired coagulation and thrombus formation.

Conflicting results have been described in terms of flow-mediated vasodilation and endothelial function following long-term RVE interventions: improvements^62^ or no significant changes^63^ have been reported. In any case, no study observed impairments of flow-mediated vasodilation or endothelial dysfunction following WBV or RVE. An improved reactive hyperemia (index of microvascular function) was observed by Beijer et al.^61^ by NIRS. The same authors described an increased number of capillaries around muscle fibers following long-term RVE intervention, as well as higher levels of total (oxygenated + deoxygenated) hemoglobin in skeletal muscle, determined by NIRS at rest, during and after exercise^61^. Other indices of cardiovascular function were observed to be improved following RVE, also in comparison with resistive exercise alone, such as an increased resting diameter and reduced arterial wall thickness^63^, improvements of indices of pulse wave reflection and cardiac pulsatile load^62^.

This current review identifies the need for further studies dedicated to investigating the effects of WBV and RVE on both clotting and thrombosis formation in healthy participants. Precisely, controlled, reproducible studies should be adopted in the future, incorporating larger sample sizes (e.g., different age of participants and different sex), with a standardization of protocol designs and data analysis. A major limitation of the present review is indeed represented by the pronounced methodological heterogeneity across studies in terms of mechanical vibration stimulus (e.g., frequency, amplitude, duration of vibration exposure and platform utilized) and measurement intervals. Future studies should also clearly consider safety issues and adverse events.

Further studies on high-risk populations, during bed rest or in long-term analogs, and eventually in-flight assessments are needed. If confirmed to be safe and effective in these conditions, WBV and RVE could be considered in the training and rehabilitation interventions to be performed in at-risk populations or in special conditions, or with the aim of preventing or attenuating the muscular and cardiovascular deconditioning associated with spaceflights, permanence on planetary habitats and ground-based simulations of microgravity. As for other types of exercise, WBV and RVE could help preventing muscle atrophy and sarcopenia, increase the muscle pump effect, induce shear stress, improve endothelial function, prevent venous stasis, inappropriate aggregation and coagulation. An obvious advantage would be represented by the fact that these interventions could be implemented reasonably easily during spaceflights and/or permanence on planetary habitats.

In summary, within the substantial limitations described above, the available evidence identified by the present systematic review suggests neutral or potentially positive effects of short- and long-term intervention with WBV and RVE on variables related to blood coagulation, fibrinolysis, inflammatory status, oxidative stress, cardiovascular, microvascular and endothelial functions. No significant warning signs towards an increased risk of undesired coagulation and venous thrombosis were identified. Although it is not possible at this stage to derive firm recommendations from the existing knowledge, mainly due to the lack of coherence in end-points across studies, the lack of any reported clotting events, despite therapeutic and leisure applications of vibration in many countries worldwide, provides some confidence.

### Supplementary information

Reporting Summary

## Data Availability

All relevant data are presented in the manuscript. Data not shown are available from the corresponding author upon request.
